# Comments in “Impact of an on-Call Specialist Aortic Rota Implementation in Acute Type an Aortic Dissection on Outcomes and Repair Complexity: A Retrospective Cohort Study”

**DOI:** 10.1093/icvts/ivag014

**Published:** 2026-02-04

**Authors:** Ankur Sharma, Varshini Vadhithala, Arun Kumar, Sushma Verma, Sushma Narsing Katkuri

**Affiliations:** Department of Anatomy, School of Medical Sciences and Research, Sharda University, Greater Noida 201306, India; Dr D. Y. Patil Medical College, Hospital and Research Centre, Dr D. Y. Patil Vidyapeeth, Deemed-to-be-University, Pimpri, Pune 411018, India; Faculty of Pharmaceutical Sciences, Graphic Era Hill University, Dehradun 248002, India; Centre for Promotion of Research, Graphic Era Deemed University, Dehradun 248002, India; Department of Pharmaceutics, Noida Institute of Engineering & Technology (Pharmacy Institute), Greater Noida 201306, India; Department of Community Medicine, Malla Reddy Institute of Medical Sciences, Malla Reddy Vishwavidyapeeth, Suraram, Hyderabad 500055, India

Dear Editor,

We read with interest the authors’ recent article on the implementation of an on-call specialist aortic rota for acute type A aortic dissection and commend them for assembling a sizeable single centre cohort and for attempting to address confounding through multivariable regression and inverse probability weighting.^1^

First, the study is an uncontrolled before-and-after comparison embedded in a broader service redesign that included protocolized operative strategies, perfusion techniques, and perioperative pathways. In such circumstances, changes in outcomes over time may reflect co-interventions, learning curves, or shifts in referral patterns rather than the rota alone. Methodological guidance on non-randomized intervention studies highlights the high risk of confounding and secular trend bias in uncontrolled before–after designs and recommends cautious causal interpretation of their findings.^2^^,^^3^ Although the authors appropriately avoid strong causal language, readers may still overinterpret the association as primarily attributable to the rota.

Second, the covariate adjustment strategy could be more clearly justified. The mortality model adjusted only for age and previous cardiac surgery, despite baseline differences or near differences in important prognostic factors such as tear location and indicators of haemodynamic instability. The inverse probability weighting analysis appears to use a different set of pretreatment covariates. Contemporary causal inference frameworks emphasize specifying a clear estimand and selecting covariates on the basis of subject matter knowledge and causal structure, rather than relying mainly on univariable *P*-value thresholds or convenience sets.^4^^,^^5^ Limited or inconsistent adjustment increases the potential for residual confounding, especially when organizational changes may influence which patients are referred and accepted over time.

Third, several aspects of reporting would benefit from clarification. In Table 1, the mean preoperative creatinine appears numerically higher in the post rota group, yet the *P* value suggests no statistically significant difference; specifying whether creatinine was skewed and which statistical test was used would help readers assess baseline comparability. In Table 2, crude tracheostomy rates (20 % vs 15%, *P* = .167) differ from the proportions highlighted in the abstract and from the adjusted association reported in the regression model, which may cause confusion about which estimates are crude and which are adjusted. In Table 3, the coefficients for cardiopulmonary bypass and cross-clamp times are labelled as odds ratios, although they appear to represent mean differences in minutes; relabelling them would avoid misinterpretation of effect magnitude.

These comments do not detract from the important clinical message that structured specialist rotas may be associated with improved short-term outcomes in complex aortic surgery. Clarifying the analytical choices, ensuring consistent reporting and, where feasible, considering designs with concurrent controls or interrupted time series would further strengthen the evidence base on service reconfiguration in acute Type A dissection.

## Data Availability

Not applicable.
